# Trastuzumab increases pulmonary vein arrhythmogenesis through modulating pulmonary vein electrical and conduction properties via phosphatidylinositol 3-kinase signaling

**DOI:** 10.22038/ijbms.2020.44651.10432

**Published:** 2020-07

**Authors:** Jun-Hei Chang, Chen-Chuan Cheng, Yen-Yu Lu, Yao-Chang Chen, Shih-Ann Chen, Yi-Jen Chen

**Affiliations:** 1 Department of Medical, Tri-Service General Hospital Songshan Branch, National Defense Medical Center, Taipei, Taiwan; 2 Department of Biomedical Engineering, National Defense Medical Center, Taipei, Taiwan; 3 Department of Cardiology, Chi-Mei Medical Center, Tainan, Taiwan; 4 Division of Cardiology, Department of Internal Medicine, Sijhih Cathay General Hospital, New Taipei City, Taiwan; 5 School of Medicine, College of Medicine, Fu-Jen Catholic University, New Taipei City, Taiwan; 6 Heart Rhythm Center and Division of Cardiology, Department of Medicine, Taipei Veterans General Hospital, Taipei, Taiwan; 7 Cardiovascular Research Center, Wan Fang Hospital, Taipei Medical University, Taipei, Taiwan;; 8 Graduate Institute of Clinical Medicine, College of Medicine, Taipei Medical University, Taipei, Taiwan

**Keywords:** Atrial fibrillation, Electrophysiology, PI3 Kinase, Pulmonary vein, Trastuzumab

## Abstract

**Objective(s):**

Drug-induced atrial fibrillation (AF) is considered an adverse effect of chemotherapeutic drugs. AF is a crucial risk factor for stroke, heart failure, myocardial infarction, and mortality. Pulmonary veins (PVs) are considered triggers inducing AF, and the sinoatrial node (SAN) may modulate PV activity and participate in AF genesis. AF was associated with early discontinuation of trastuzumab in patients with breast cancer. However, whether trastuzumab directly modulates the electrophysiological characteristics of PV and SAN remains unclear.

**Materials and Methods:**

ECG and conventional microelectrode system were used to record rabbit heart rhythm *in vivo* and electrical activities *in vitro* from isolated SAN, PV, and SAN-PV preparations.

**Results:**

Trastuzumab reduced the beating rate in isolated PV and SAN preparations at 1, 10, and 30 μM (particularly in isolated SAN preparations) and induced burst firings in isolated PV preparations at 10 μΜ. In addition, trastuzumab (10 μM) induced SAN-PV conduction block and burst firings, which were blocked by wortmannin (a PI3K inhibitor, 100 nM). Similarly, ECG recordings showed that acute intravenous administration of trastuzumab (10 mg/kg) reduced rabbit heart rates.

**Conclusion:**

Trastuzumab increased PV arrhythmogenesis through interfering with PI3K signaling, which may contribute to the genesis of AF.

## Introduction

Atrial fibrillation (AF) is the commonest sustained arrhythmia in clinical practice and is a crucial risk factor for stroke, heart failure, myocardial infarction, and mortality (1-5). Through multiple mechanisms, cancer is reported to be an important risk factor for AF (6, 7), whereas the prevalence of a concomitant history of cancer was reported in up to 20% of AF patients (8, 9). AF development may affect the prognosis and therapeutic effects of cancer patients (6) and become one of the critical issues during or after cancer therapy (7).

Cardiotoxicity is one of the most adverse effects of anticancer treatment (10). Drug-induced AF is considered an adverse effect of chemotherapeutic drugs, such as alkylating agents, anthracycline agents, and cancer-targeted therapies (11). Targeted therapy increases the risk of cardiotoxicity in cancer patients, which illustrates their off-target effects on the heart (12, 13). Breast cancer is the most common cancer in women worldwide (14). Trastuzumab is a humanized monoclonal antibody that selectively binds to the extracellular domain of the human epidermal growth factor receptor 2 (HER2). Therefore, trastuzumab is used to improve outcomes in early and advanced HER2-positive breast cancer (15-17). Emerging evidence indicates that trastuzumab may play a role in the pathological process of drug-induced cardiotoxicity (18-25) AF was associated with a high risk of heart failure or cardiomyopathy in breast cancer patients after adjuvant trastuzumab therapy (26) and with early discontinuation of trastuzumab in patients with breast cancer (27). The incidence of AF in patients with breast cancer receiving trastuzumab was approximately 1.2% across different studies (27-29). The HER-2-induced phosphoinositide 3-kinase (PI3K) signaling pathway was involved in the pathogenesis of cancer (30, 31). Decreased cardiac PI3K activity increases the risk of AF (32). In addition, the risk factors of AF, including aging, obesity, and diabetes have been shown to be associated with depressed/defective PI3K signaling.(33-35) In addition, trastuzumab markedly increased apoptotic cells and cardiac fibrosis in animal experiments (36), which may induce cardiac dysfunction and AF genesis (37). However, it is not clear whether trastuzumab has direct effects on cardiac electrophysiology, leading to the genesis of AF.

 AF may originate from the triggered activity of ectopic foci in the pulmonary veins (PVs) (38, 39). The PVs contain complex histological components with vascular structures and cardiomyocytes and play a critical role in the genesis and maintenance of AF (40). Additionally, the sinoatrial node (SAN) electrical activity could modulate PV arrhythmogenesis through overdrive suppression of PV spontaneous activity. The electrical competition between SAN and PVs may determine the risk of AF occurrence (41). Therefore, the purpose of this study was to investigate whether trastuzumab increases PV arrhythmogenesis through modulating SAN and PV electrical properties and to evaluate its underlying mechanisms.

## Materials and Methods

***Animal and tissue preparations***

The experiments in this study conform to requirements of the institutional Guide for the Care and Use of Laboratory Animals and were approved by a local ethics review board (IACUC-18-127). Male rabbits (2.0–3.0 kg) were anesthetized with overdose of isoflurance (5% in oxygen) from a precision vaporizer. Adequacy of anesthesia was confirmed by the lack of corneal reflex and monitoring response to pain stimuli by scalpel tip. A mid-line thoracotomy was performed and the hearts were rapidly removed (42). SAN with the right atrium and the right superior PV with the LA were isolated for the experiments at the intact PV-SAN tissue preparations. For isolated PV or SAN experiments, the PVs were dissected by an incision along the mitral valve annulus, extending from the coronary sinus to the septum, and the SANs were dissected from the right atrium and superior vena cava in Tyrode’s solution composed of NaCl 137 mM, KCl 4 mM, NaHCO_3 _15 mM, NaH_2_PO_4_ 0.5 mM, MgCl_2 _0.5 mM, CaCl_2_ 2.7 mM, and dextrose 11 mM. 

***Electropharmacological experiments***

A conventional microelectrode system was used to record the electrical activities and conduction properties in isolated rabbit PV, SAN, and intact PV-SAN preparations, as described previously (43). One end of the preparations was pinned with needles to the bottom of a tissue bath. The other end was connected to a Grass FT03C force transducer with a silk thread. The adventitial or epicardial side of the preparations faced upward. The tissue strips were superfused at a constant rate (3 ml/min) with Tyrode’s solution saturated with a 97% O_2_-3% CO_2_ gas mixture. The temperature was maintained at 37 ^°^C, and the preparations were allowed to equilibrate for 1 hr before electrophysiological assessment. Transmembrane action potentials were recorded by using machine-pulled glass capillary microelectrodes.

The transmembrane action potentials (APs) of the isolated PV, isolated SAN, or SAN-PV preparations were recorded using machine-pulled glass capillary microelectrodes filled with KCl 3 M, and tissue preparations

were connected to a model FD223 electrometer (World Precision Instruments, Sarasota, FL, USA) under a tension of 150 mg. Electrical and mechanical events were simultaneously displayed on a Gould 4072 oscilloscope and Gould TA11 recorder (Gould Instruments, Cleveland, OH, USA). Signals were recorded with DC coupling and a 10-KHz low-pass cutoff frequency ﬁlter using a data acquisition system. Different concentrations of trastuzumab (Herceptin**®, **0.1, 1, 10, and 30 μM, F. Hoffmann-La Roche, Basel, Switzerland) were sequentially superfused for at least 20 min to test pharmacological responses of the isolated PV, isolated SAN, or SAN-PV preparations. PVs or SANs with spontaneous activity were defined as the constant occurrence of spontaneous APs with no electric stimulus. To study the mechanisms of trastuzumab, wortmannin (PI3K inhibitor, 100 nM) was administered to PV in the presence or absence of trastuzumab (10 μM). Burst firing was defined as accelerated spontaneous activity that was faster than the basal beating activity, with the characteristics of sudden onset and termination. Delayed afterdepolarizations were defined as the presence of a spontaneous depolarization of the impulse after full repolarization (44). 

***Electrocardiogram measurement***

Every ECG recording was taken under deep sedation induced by overdose of isoflurance (5% in oxygen) from a precision vaporizer. The animal was kept in supine position, and the recording electrodes were placed subcutaneously on four limbs. Digital surface ECG was taken simultaneously from all limb leads and continuously recorded for at least 20 min after receiving sequentially intravenous administration of trastuzumab (0.1 mg/kg and 10 mg/kg). The data was saved for further analysis, which was conducted by utilizing software included with the PageWriter Trim III (Philips Medical Systems, MA, USA). Measurement was taken from limb lead II with the highest quality of recording, and the paper speed of ECG recording was 25 mm/sec and calibration of 1 mV/10 mm. The P-R interval is measured from the beginning of the upslope of the P wave to the beginning of the QRS wave. The QT interval was defined as the time from the onset of the QRS complex to the end of the T wave, while the RR interval measured the time between the peaks of QRS complexes from two consecutive heart beats. The QT interval and RR interval were subsequently used for calculating the corrected QT interval. The rate-corrected QT interval (QTc) was calculated by using Bazett’s formula [QTc = QT/RR^1/2^].

***Statistical analysis***

All continuous parameters were expressed as ± the standard error of the mean (SEM). One-way analysis of variance (ANOVA) with a Duncan *post-hoc* test was used to compare differences between the groups. Nominal variables were compared using Chi-squared analysis with Fisher’s exact test. A *P*-value of <0.05 was considered statistically significant.

## Results

***Effects of trastuzumab on PV and SAN spontaneous activity***

**Figure 1:**
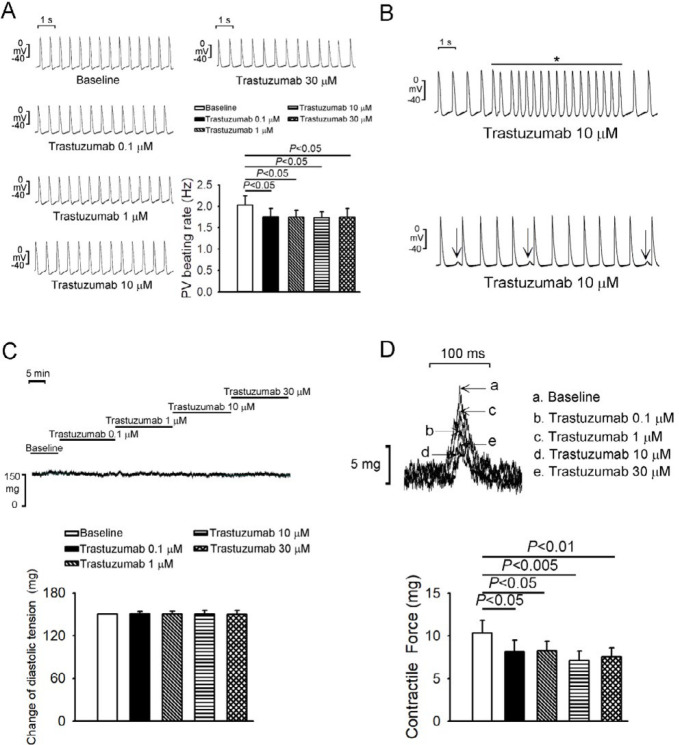
Effects of trastuzumab on pulmonary vein (PV) spontaneous activity, vessel tone, and contractility. (A) Tracings and average data of action potentials before and after different concentrations (0.1 μM, 1 μM, 10 μM, and 30 μM) of trastuzumab on PV spontaneous activity (N=7). (B) Representative recordings of the effect of trastuzumab (10 μM)-induced delayed afterdepolarization or burst firing in PV preparations. (C) Tracing and average data of diastolic tension before and after different concentrations (0.1 μM, 1 μM, 10 μM, and 30 μM) of trastuzumab on PVs. (D) Tracings and average data of contractile force before and after different concentrations (0.1 μM, 1 μM, 10 μM, and 30 μM) of trastuzumab on PVs

**Figure 2:**
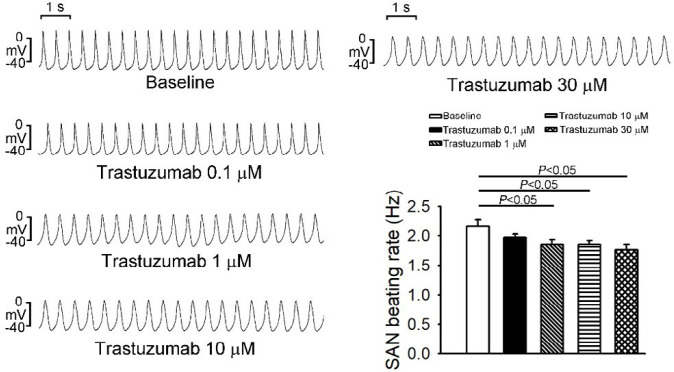
Effects of trastuzumab on sinoatrial node (SAN) spontaneous activity. Tracing and average data of action potentials before and after different concentrations (0.1 μM, 1 μM, 10 μM, and 30 μM) of trastuzumab on SAN spontaneous activity (N=6)

Figure 1A shows that trastuzumab at 0.1, 1, 10, and 30 μM reduced PV spontaneous activity by 13%, 14%, 16%, and 14%, respectively. Trastuzumab induced burst firings with a rate of up to 17 Hz in 4 (57%, *P*<0.05) of 7 PV preparations (*P*<0.05) at 10 or 30 µM but not at 0.1 and 1 µM (Figure 1B). Additionally, trastuzumab reduced PV contractility but did not change PV diastolic tension. Compared to baseline, trastuzumab significantly reduced SAN spontaneous activity by 9%, 15%, 15%, and 18% at the concentrations of 0.1, 1, 10, and 30 μM, respectively (Figure 2). Trastuzumab did not induce burst firing in any SAN preparations with different concentrations.

***Effects of trastuzumab on PV and SAN electrical and conduction properties***

**Figure 3:**
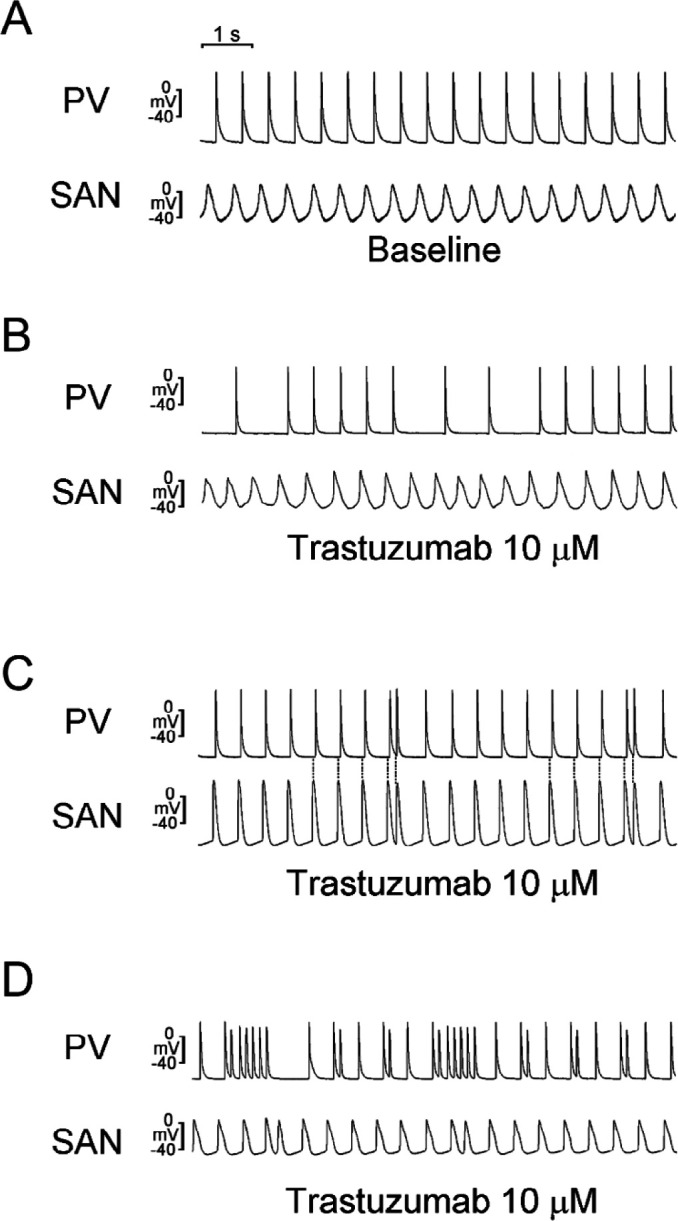
Effects of trastuzumab on sinoatrial node (SAN)-pulmonary vein (PV) spontaneous activity. Representative recordings of (A) baseline, (B) conduction block, (C) triggered activity, and (D) burst firing in SAN-PV preparations with trastuzumab (10 μM)

As shown in Figure 3, trastuzumab (10 μΜ) produced SAN-PV conduction block in 5 of 8 preparations (*P*<0.05) and induced the occurrence of trigger activity or PV burst firings in 7 of 8 tissue preparations (*P*<0.005).

***Effect of trastuzumab on the electrical activity of SAN-PV preparations treated with wortmannin***

**Figure 4:**
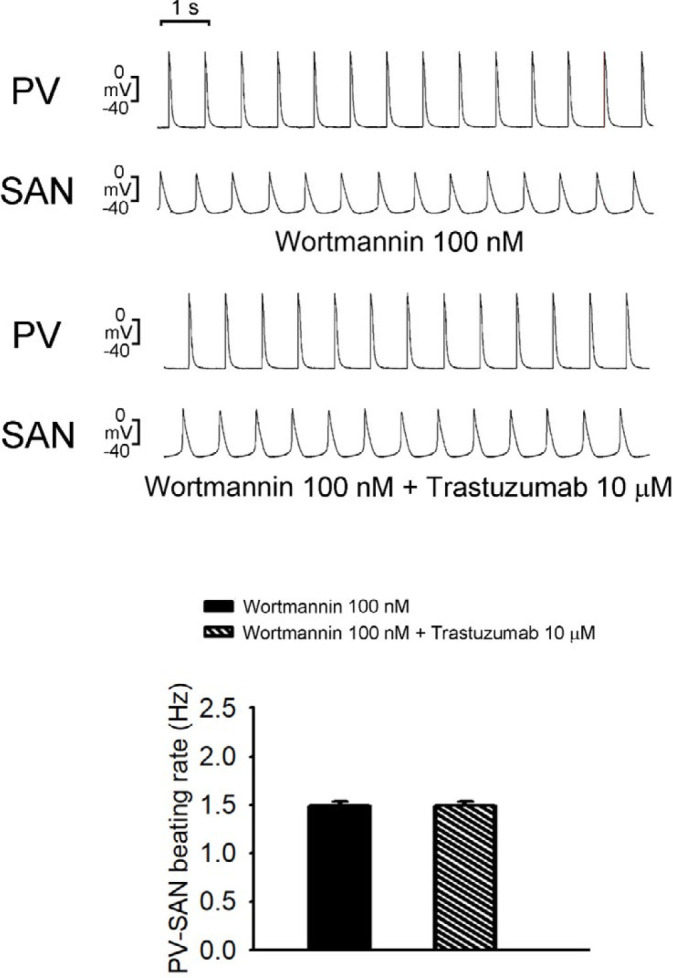
Effects of trastuzumab on wortmannin-treated sinoatrial node (SAN)-pulmonary vein (PV) spontaneous activity. Tracings and average data of spontaneous activity in wortmannin-treated SAN-PV preparations (N=8) with trastuzumab (10 μM)

In the presence of wortmannin (100 nΜ), trastuzumab (10 μΜ) did not change the rate of spontaneous activity of SAN-PV preparations. Additionally, trastuzumab (10 μΜ) neither induced trigger activity nor produced SAN-PV conduction block (Figure 4).

***In vivo electrocardiogram measurements***

**Figure 5:**
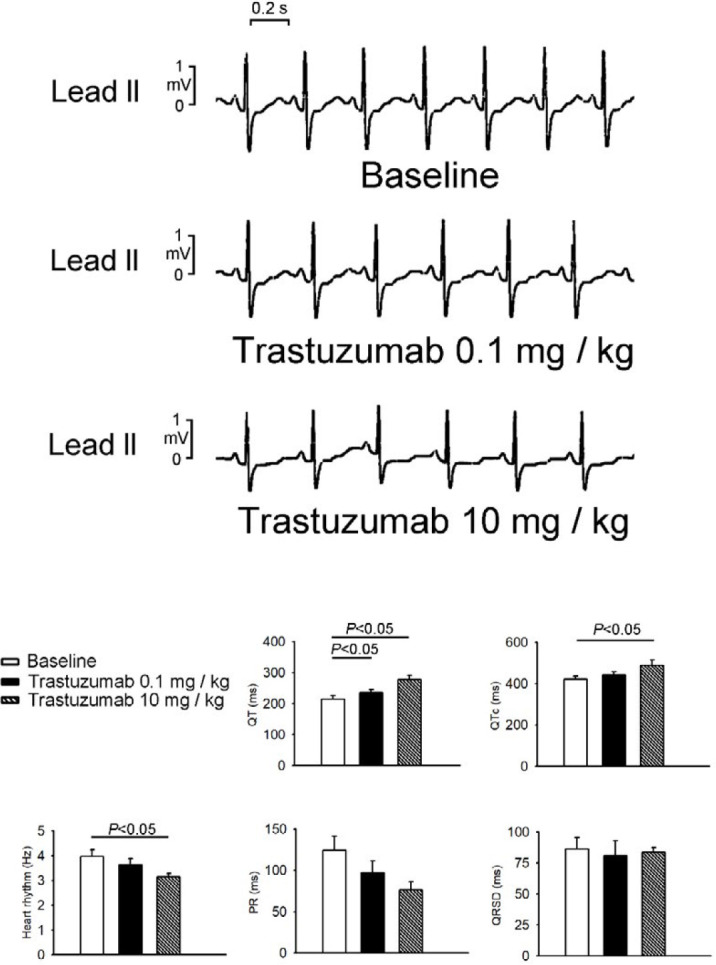
Effects of trastuzumab on rabbit heart rhythm in vivo. Upper panel showed representative ECG tracings (lead II) before and after trastuzumab (0.1 mg/kg) and trastuzumab (10 mg/kg). Lower panel showed the average data (N=4) of different ECG parameters before and after different doses of trastuzumab

As shown in Figure 5, acute intravenous administration of trastuzumab (10 mg/kg) reduced rabbit heart rates. In addition, trastuzumab (0.1 mg/kg and 10 mg/kg) prolonged QT interval, and trastuzumab (10 mg/kg) prolonged QTc. However, trastuzumab did not change PR interval or QRS duration.

## Discussion

Although cardiotoxicity is one of the well-known toxicities caused by trastuzumab, the present study, for the first time, showed that trastuzumab can induce PV arrhythmogenesis. The concentrations of trastuzumab in circulation were reported to be approximately 0.23–0.83 μΜ in patients taking a fixed 600 mg subcutaneous dose of trastuzumab (45). Since trastuzumab reduced PV and SAN electrical activity or contractility at a concentration as low as 0.1 µM, the concentration of trastuzumab used in this study may be clinically relevant. Syncope with documented bradycardia from SAN dysfunction was reported in one patient treated with trastuzumab (46). We found that trastuzumab reduced both PV and SAN spontaneous activity, and induced conduction abnormalities. Similarly, our *in vivo* experiments also showed that trastuzumab at high dose (10 mg/kg) reduced rabbit heart rate. It is suggesting the high proarrhythmic risk of trastuzumab may induce AF occurrence due to increasing PV burst firing and loss of SAN modulation on PV spontaneous activity (41). However, trastuzumab induced burst firing in isolated PV preparations and produced SAN-PV conduction block at relatively higher concentrations (10 and 30 µM), which may be supraphysiological and not clinically relevant. 

The PI3K pathway is a critical regulator of cardiac protection under stress conditions (47). Increased PI3K activity reduces atrial fibrosis and improves cardiac conduction, whereas reduced PI3K activation increases the susceptibility to AF (32, 48). PI3K signaling was reported to up-regulate gene expression of sodium channel subunits (49). Wortmannin (100 nM) has been shown to completely suppress PI3K activity (50). In the presence of wortmannin (100 nM), trastuzumab (10 μM) did not reduce SAN-PV spontaneous activity, suggesting that wortmannin blocked the inhibitory effect of trastuzumab. Therefore, trastuzumab (10 μM) may decrease SAN-PV spontaneous activity through its inhibitory effects on PI3K. PI3K modulation by trastuzumab impairs conduction and SAN automaticity and increases PV arrhythmogenesis. Trastuzumab exerts a negative chronotropic effect that reduces SAN spontaneous activity. PI3K critically affects cardiac contractility. Cardiac-specific PI3Kα overexpression results in enhanced contractility (51). Pharmacological inhibition of PI3K by trastuzumab reduces calcium currents and contractility (52), which play a role in drug-induced cardiotoxicity. Since the time considered for trastuzumab exposure to tissue (20 min) is too short to act via gene transcription regulation, posttranslational modification with protein phosphorylation by trastuzumab was hypothesized to contribute to its acute biological effects. Previous study has shown that characteristic phosphorylation times for the receptors and downstream kinases were within minutes (53). Therefore, our findings suggested that trastuzumab increased PV arrhythmogenesis through interfering with PI3K signaling, leading to phosphorylation and activation of downstream PI3K target molecules. Moreover, we found that trastuzumab (from 0.1 to 30 µM) did not dose-dependently reduce SAN or PV spontaneous activity. Although the mechanisms underlying these results are not elucidated, it is hypothesized that PI3K substrate may be fully phosphorylated by trastuzumab at low concentrations, leading to its non-dose dependent electrophysiological effects (54). Previous study also has shown that trastuzumab may not have dose-dependent effects at high concentrations (55). The concentrations of trastuzumab used in this study are much higher than the therapeutic range (45). However, there are not any molecular experiments regarding AF and trastuzumab effect in this study. The spatial–temporal distribution of trastuzumab-induced posttranslational modification in cells and tissues remains unclear.

## Conclusion

Trastuzumab can directly modulate SAN and PV electrical and conductive properties and induce PV arrhythmogenesis via PI3K signaling, which may contribute to the occurrence of AF in trastuzumab-treated patients. 
